# Cost-utility of vitamin D supplementation to prevent acute respiratory infections in children

**DOI:** 10.1186/s12962-023-00433-z

**Published:** 2023-04-06

**Authors:** Jefferson Antonio Buendía, Diana Guerrero Patiño

**Affiliations:** Research group in Pharmacology and Toxicology ”INFARTO”, Department of Pharmacology and Toxicology, University of Antioquia, Medellin, Colombia

**Keywords:** Health economics, Public health, Healthcare, Colombia, Corticosteroids

## Abstract

**Introduction:**

Increasing evidence has demonstrated the effectiveness and safety of vitamin D supplementation to prevent acute respiratory infections in children. More economic evaluations incorporating the new evidence and in the pediatric population are needed to know the efficiency of this treatment. This study aimed to determine the cost-utility of vitamin D supplementation to prevent acute respiratory infections in pediatric patients.

**Methods:**

A decision tree model was used to estimate the cost and quality-adjusted life-years (QALYs) of vitamin D supplementation in healthy school children between 1 and 16 years. Multiple sensitivity analyses were conducted. Cost-effectiveness was evaluated at a willingness-to-pay (WTP) value of $19,000.

**Results:**

The base-case analysis showed that vitamin D supplementation was associated with lower costs and higher QALYs than strategy without this supplementation. The QALYs per person estimated in the model for those treatments were 0,99 with vitamin D supplementation and 0,98 without vitamin D supplementation. The total costs per person were US$ 1354 for vitamin D supplementation and US$ 1948 without vitamin D supplementation. This position of absolute dominance of vitamin D supplementation makes it unnecessary to estimate the incremental cost-effectiveness ratio.

**Conclusion:**

In conclusion, our study shows that Vitamin D supplementation is a cost-effective strategy to prevent ARI in pediatric patients, from a societal perspective.

## Introduction

In the last decades, the number of episodes of acute respiratory infections in young children has decreased by 22%; however, acute respiratory infections (ARI) remain in the top ten of the most common causes of death in children worldwide [1]. Improvements in rates of exclusive breastfeeding, nutrition, complete immunization, and reduction of indoor air pollution and pediatric HIV across all regions between 2000 and 2015 explain the reduction of several cases of ARI [1, 2]. Also, increases in access to health services have been reflected in an 187% increase in hospital admissions for pneumonia [1]. This increase generates an excessive economic burden for health systems, especially in countries or settings with low health resources.

1,25(OH)2D3 is a key component of response in immune cells in response to pathogens. In macrophages infected, the expression of several cytokines, including interleukin 1β (IL-1β), a core component of innate immune responses, and the neutrophil chemokine IL-8/CXCL8 are induced in presence of 1,25(OH)2D3 [3]. Also, 1,25(OH)2D3 contributes to the suppression of peripheral inflammatory T cell responses and enhanced development of T-regulatory (Treg) cells [4]. This can explain that in vitamin D-supplemented patients the antimicrobial activity in pulmonary surface airway fluid is significantly higher [5].

A systematic review and meta-analysis of 7434 pediatric patients found high levels (64% of frequency) of 25OHD deficiency (47% < 50 nM) in children with sepsis (p < 0.0001), also associated with increased mortality (OR 1.81; 95% CI 1.24 to 2.64; p = 0.002, I2 = 25.7%) [6]. Also, a recent systematic review and meta-analysis of 48 488 participants (aged 0–95 years) in 43 studies found a significantly lower proportion of participants in the vitamin D supplementation group had one or more ARIs (14 332 [61·3%] of 23 364 participants) than in the placebo group (14 217 [62·3%] of 22 802 participants), with an OR of 0·92 (95% CI 0·86–0·99; 37 studies; *I*²=35·6%) [7].

There is no consensus among the different clinical practice guidelines on recommending Vitamin D to prevent ARI in children. However, with recent evidence, these recommendations may change. The most recent meta-analysis published in 2021 evidenced that in children years there is a reduction in risk of ARI (0·71 (0·57–0·90]; 15 studies; I²=46·0%. Unfortunately, only the effectiveness and safety of this treatment have been evaluated in most clinical practice guidelines, ignoring the need for a third element such as efficiency. When efficiency is evaluated through complete economic evaluations, outcomes such as days of hospital stay, clinical or radiological cure play an important role, as does survival itself, since these are outcomes that directly impact the cost. The contribution of an economic evaluation to the current evidence lies not only in estimating whether it is cost-effective, but also in determining other outcomes that are inputs for estimating the impact of such an intervention at the public health, such as the cost-savings per patient treated with this supplementation. Also, to evaluate if there is any effect-modifying that can estimate under what conditions if supplementation is efficient. In synthesis, more economic evaluations incorporating the new evidence and in the pediatric population are needed to know the efficiency of this treatment. More economic evaluations incorporating the new evidence and in the pediatric population are needed to know the efficiency of this treatment. The objective of the present study was to determine the cost-utility of vitamin D supplementation to prevent ARI in pediatric patients.

## Materials and methods

### Base case

**Fig. 1:**
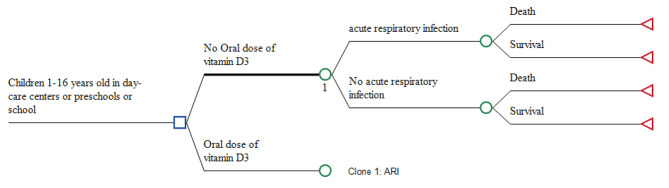
Markov model

A decision tree model was used to estimate the cost and quality-adjusted life-years (QALYs) of vitamin D supplementation compared without vitamin D supplementation, Fig. 1.

We searched the Cochrane Library, Embase, and PubMed in order to retrieve metanalysis about the effect of vitamin D supplementation in the prevention of ARIs from inception to November 2022. Keywords used for searching were as follows: “vitamin D”, “respiratory tract infections,” and “meta-analysis”. Out of a total of 104 articles initially searched, and after excluding duplicated articles or articles did not reported results in pediatric patients, 7 metanalysis were reviewed [6–12]. The meta-analysis of Jolliffe et al. [7] were selected to extract the relative risk of this decision tree model because it was not only the most recently published, but also contained data from previous studies. This meta-analysis evaluated the efficacy and safety of vitamin D supplementation (400–1000 IU daily for 6–12 months) in 15 RCTs with 11,871 healthy school children between 1 and 16 years [7]. ARI events in this meta-analysis included upper respiratory infection, lower respiratory infection, or both. In this study, a protective effect of vitamin D supplementation on the risk of having one or more ARIs was observed in participants aged 1.00–15.99 years (0·71 [0·57–0·90]; I²=46·0%), without difference between participants in the incidence of serious adverse events of any cause, death due to any cause, hypercalcemia, or renal stones.

It was decided to use a decision tree model because we are going to model interventions that have distinct outcomes that can be measured at a specific time point, such in this case cost and QALY associated with the incidence of ARI in the next six months after of vitamin D supplementation in healthy school children between 1 and 16 years. In our economic model, a tree decision, and according to the natural history, four health states were defined: “death after acute respiratory infection”, “Survival after acute respiratory infection”, “Survival without acute respiratory infection”, and “death for all causes”. Then, in both decision nodes, there are two possibilities that the patient dies or survives after of episode of ARI. The only difference between the two decision branches is the probability of ARI at 6 months. This probability in the on the branch with vitamin D supplementation is lower than in the branch without this supplementation because it was multiplied by the relative risk of this intervention as detailed later. The time horizon defined was six months. Given the short time horizon, no discount rates were applied to costs or QALYs. Cost-effectiveness was evaluated at a willingness-to-pay (WTP) value of $19,000 using the World Health Organization’s (WHO) recommendation for choosing cost-effective interventions based on a country’s gross domestic product (GDP) per capita (1 to 3 times country`s gross domestic product per capita) [13].

Data on transition probabilities were obtained from local data reported by national surveillance of ARI, national vital statistics [14–16], Table 1. Utilities were extracted from a utility assessment study of parent preferences for pediatric health outcomes made in 4016 participants [17]. Since utilities and relative risks do not come from the Colombian population, they were subjected to probabilistic sensitivity analysis as detailed below as recommended by Consolidated Health Economic Evaluation Reporting Standards (CHEERS) Statement [18]. We did this analysis from a societal perspective (including direct and indirect costs). All direct and indirect costs for each health state defined in the model were extracted from a previously published Colombian study in children with pneumonia [19]. This study estimates the direct medical costs and indirect non-medical costs of pneumonia in children under 5 years, based on information obtained from 275 patients hospitalized using the databases of the Individual Registry of Services Provision and Sufficiency and a survey constructed, validated, and applied to parents, mothers, and caregivers of children hospitalized by these events.

**Table 1:** Model inputs

Model input		Base case value		Distribution		
**Probabilities**						
ARI		**0.22**		β(SD: 0.005)		
Mortality ARI		**0.0001**		β(SD: 0,000029)		
Mortality All-Causes		**0,0008**		β(SD: 0,00022)		
**Disutility**						
ARI		**0.06**		β(SD: 0.015)		
**Cost/day**						
ARI (US$)		**880**		Γ (SD: 222)		
Vit D3 400 UI/daily (US$)		**0,08**		Γ (SD: 0.02)		
**Vit D3 effectiveness**						
Relative risk of reduction of ARI		**0.71**		LogN(SD: 0.11)		

ARI = acute respiratory infection

Drug costs were taken from the National Drug Price Information System (SISMED, 2020) [20]. All cost costs were transformed to 2020 costs using official inflation data in Colombia. We used US dollars (Currency rate: US$ 1.00 = COP$ 3,000) to express all costs in the study [15].

### Sensitivity analysis

We conduct a one-way sensitivity presenting these results in the tornado diagram. Probabilistic sensitivity analysis was also performed. For this purpose, random sampling was performed from each of the parameter distributions. We used the beta distribution for relative risk and utilities and the gamma distribution for costs. For each treatment strategy, we calculated the expected costs and QALYs using the combination of all parameter values in the model. To do this calculation, a second-order Monte Carlo simulation with 10,000 replications of each parameter was made: resulting in the expected cost-utility for each treatment strategy. To represent decision uncertainty, we plot the cost-effectiveness and acceptability frontiers. Microsoft Excel® was used in all analyses.

## Results

The base-case analysis showed that vitamin D supplementation was associated with lower costs and higher QALYs than strategy without this supplementation. The QALYs per person estimated in the model for those treatments were 0,99 with vitamin D supplementation and 0,98 without vitamin D supplementation. The total costs per person were US$ 1354 for vitamin D supplementation and US$ 1948 without vitamin D supplementation. This position of absolute dominance of vitamin D supplementation makes it unnecessary to estimate the incremental cost-effectiveness ratio, Table 2.

**Table 2:** Cost effectiveness analysis

Strategy	Cost (US$)	Diff ($)	QUALYs	Diff (QALYs)	NMB($)
Vit D3	1354		0,99		18,791
Non-Vit D3	1948	594	0,98	0,01	18,733

### Sensitivity analysis

**Fig. 2:**
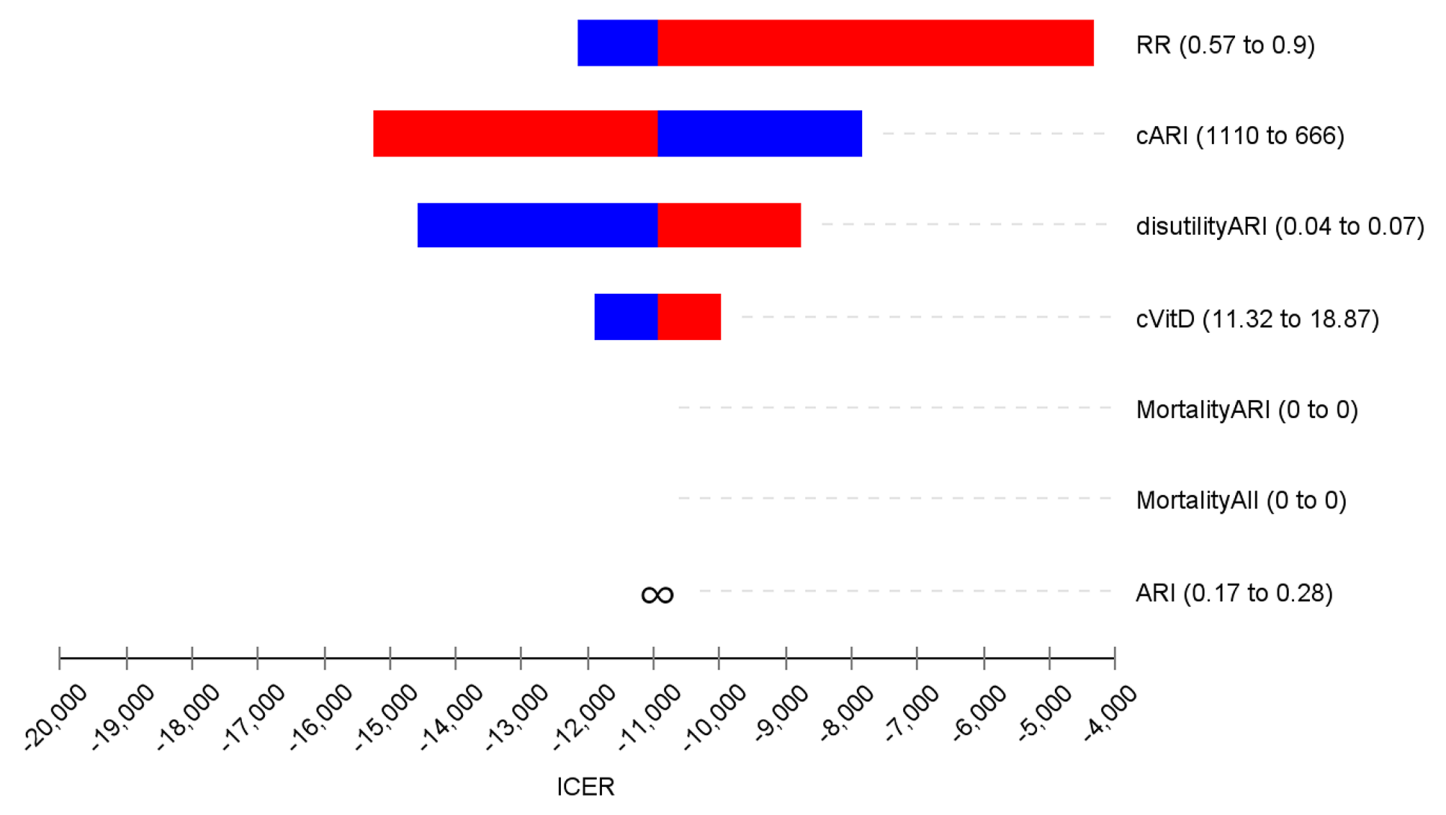
Tornado diagram RR : Relative risk ARI = acute respiratory infection

**Fig. 3:**
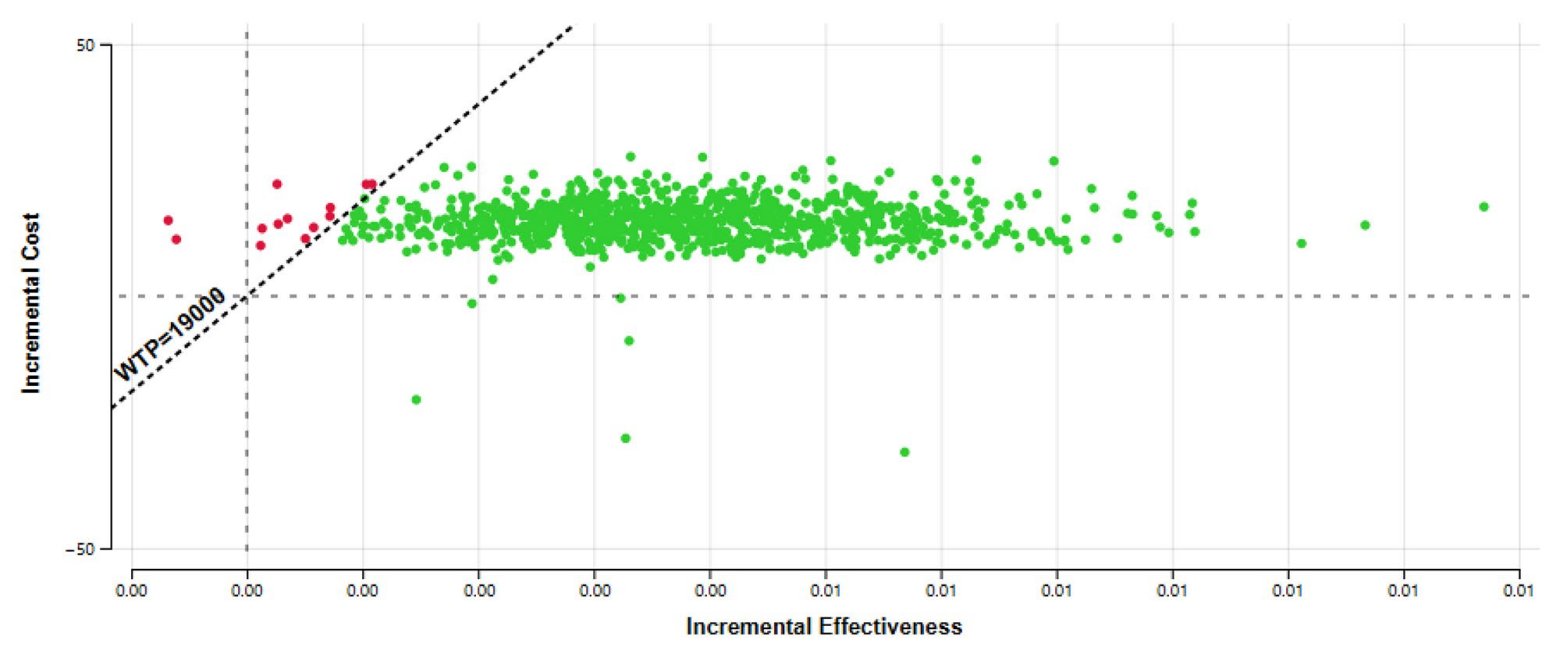
Cost effectiveness plane

**Fig. 4:**
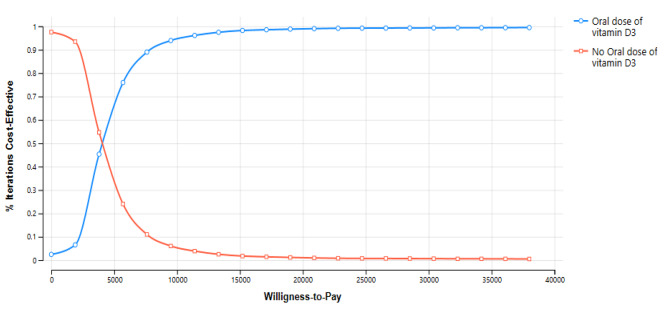
Acceptability Curve

In the deterministic sensitivity analyses, our base-case results were robust to variations in utilities, transition probabilities, relative risk, and cost; Fig. 2. The results of the probabilistic sensitivity analysis are graphically represented in the cost-effectiveness plane, Fig. 3. This scatter plot shows that 96% of simulations were graphed in quadrant 1 (high cost, high QALYs) below WTP. In the cost-effectiveness acceptability plot, vitamin D supplementation becomes a cost-effectiveness alternative if WTP is equal to or higher than US$ 4000 Fig. 4.

## Discussion

Our economic evaluation shows that vitamin D supplementation is cost-effective to prevent ARI in pediatric patients. Evaluating treatments to reduce costs and optimize health resources is a priority for all health systems, especially in ARI, which, due to their frequency, generate a high economic burden. In our study, vitamin D supplementation had a chance of being cost-effective between 98 and 75% at a WTP between 1 and 3 GBD per capita in Colombia, respectively; supporting the implementation of this strategy generates savings of US$ 519 per patient. This potential savings is not negligible, even more so if we take into account that there are more than 3 million cases of ARI in our country every year.

To our knowledge, this is the first economic evaluation of vitamin D supplementation in the prevention of ARI. The efficiency of some interventions has been evaluated previously to prevent ARI. Sakai et al., evaluate the cost-effectiveness of gargling to prevent upper respiratory tract infection during an RCT in 252 healthy adults volunteers [21]. Gargling (Gargle with approximately 20 ml of water or povidone-iodine for about 15 s, 3 times/day) was cost-effective, after 60 days, with an increase in QALY of 0.43 and ICER of $31,800 per QALY; model that was sensible to cost of gargling and utilities. From Japan’s societal perspective, this ICER was within the range of other preventive methods such as influenza vaccination. Barber et al. evaluated the cost-effectiveness of OM-85 to prevent ARI in children 2–6 years in Mexico from a third payer perspective. OM-85 was cost-effective in preventing ARI, and also showing cost savings in over 70% of cases for direct costs with a reduction of 2.61 episodes of ARI during a follow-up of six months [22]. These results with OM-85 have been similar to those obtained in other countries such as France and Italy [23–25].

In contrast to the evidence on efficiency, there is a greater number and diversity of evaluations of the effectiveness of different interventions to prevent ARI. A Cochrane systematic review of 15 RTC shows that probiotics were better than placebo in reducing the number of participants experiencing episodes of acute URTI (least one episode: odds ratio (OR) 0.53; 95% confidence interval (CI) 0.37 to 0.76; P-value < 0.001; low-quality evidence), the mean duration of an episode of acute URTI, antibiotic use and cold-related school absence. However, the quality of the evidence was low or very low [26]. A recent systematic review of 54 studies (4851 children) judged to be of moderate quality [27, 28]; reported a statistically significant association between treatment with OM-85 and a reduction in the frequency of respiratory infections (MD -2.33; 95% CI -2.75, − 1.90; P < 0.00001) and a statistically significant reduction in fever days in the group treated with OM-85 compared to the control group was reported (MD − 2.91 days; 95% CI -3.75, − 2.07; p < 0.00001). On June 27, 2019, EMA recommended the use of medicines containing bacterial lysate only for the prevention of recurrent respiratory infections except for pneumonia [27]. Other Cochrane systematic review of six RTC with 5193 children aged from 2 months to 59 months showed that zinc supplementation reduced the incidence of pneumonia by 13% ( RR 0.87; 95% confidence interval (CI) 0.81 to 0.94, six studies, low-quality evidence) and prevalence of pneumonia by 41% (RR 0.59; 95% CI 0.35 to 0.99, one study, n = 609, low-quality evidence) [29].

There is no consensus concerning interventions to eliminate certain risk factors to prevent ARI or recurrent acute respiratory infections. Reducing exposure to damp and mold for example, is the intervention for which a good-quality systematic review supports the elimination of this risk factor for recurrent respiratory acute infections [27]. Other environmental interventions with low or very low-quality studies are also recommended, such as discouraging exposure to second and third-hand smoke and pollutants in general, in addition, to improving hand-washing as one of the best methods to reduce respiratory infections [27, 30].

Our study has some limitations. We use relative risk extracted from the literature and not estimated directly from our population. As was mentioned previously, the reliability and robustness of the results were evaluated by sensitivity analysis. The results of this economic evaluation given the base case are only applicable to children. The cost was obtained from a retrospective study published previously from Colombia and cannot exclude selection or information bias in these values. However, the ICER estimate was robust to any variation in the cost in the study. The current metanalysis do not evaluated the efficacy of vitamin D supplementation considering the baseline concentrations of 25(OH)D and there are doubt if there are differences in the effect in patients with vitamin D deficiency and normal vitamin D levels. Also, the heterogeneity of randomized controlled trials of found by other metanalysis of vitamin D supplements on ARIs is high and this can explain also the differences in the results between systematic review published [31]. However our results were robust to variations of relative risk, given confidence about possible variation on the effectiveness of this intervention.

## Conclusion

In conclusion, our study shows that Vitamin D supplementation is a cost-effective strategy to prevent ARI in pediatric patients, from a societal perspective. This evidence will undoubtedly guide policymakers toward more efficient use of health resources.

## Data Availability

Zenodo. 10.5281/zenodo.5895163.

## List of abbreviations

RR: relative risk
RTC: randomized controlled trial
OR: odds ratio
CAP: community-acquired pneumonia
WTP: willingness-to-pay
QALY: quality adjusted life years
ICER: incremental cost effectiveness ratio

## References

[1] McAllister DA, Liu L, Shi T, Chu Y, Reed C, Burrows J (2019). Global, regional, and national estimates of pneumonia morbidity and mortality in children younger than 5 years between 2000 and 2015: a systematic analysis. Lancet Glob Health.

[2] Howie SRC, Murdoch DR (2019). Global childhood pneumonia: the good news, the bad news, and the way ahead. Lancet Glob Health.

[3] Verway M, Bouttier M, Wang TT, Carrier M, Calderon M, An BS (2013). Vitamin D induces interleukin-1beta expression: paracrine macrophage epithelial signaling controls M. tuberculosis infection. PLoS Pathog.

[4] Urry Z, Chambers ES, Xystrakis E, Dimeloe S, Richards DF, Gabrysova L (2012). The role of 1alpha,25-dihydroxyvitamin D3 and cytokines in the promotion of distinct Foxp3 + and IL-10 + CD4 + T cells. Eur J Immunol.

[5] Vargas Buonfiglio LG, Cano M, Pezzulo AA, Vanegas Calderon OG, Zabner J, Gerke AK (2017). Effect of vitamin D3 on the antimicrobial activity of human airway surface liquid: preliminary results of a randomised placebo-controlled double-blind trial. BMJ Open Respir Res.

[6] Cariolou M, Cupp MA, Evangelou E, Tzoulaki I, Berlanga-Taylor AJ (2019). Importance of vitamin D in acute and critically ill children with subgroup analyses of sepsis and respiratory tract infections: a systematic review and meta-analysis. BMJ Open.

[7] Jolliffe DA, Camargo CA, Sluyter JD, Aglipay M, Aloia JF, Ganmaa D (2021). Vitamin D supplementation to prevent acute respiratory infections: a systematic review and meta-analysis of aggregate data from randomised controlled trials. Lancet Diabetes Endocrinol.

[8] Martineau AR, Jolliffe DA, Hooper RL, Greenberg L, Aloia JF, Bergman P (2017). Vitamin D supplementation to prevent acute respiratory tract infections: systematic review and meta-analysis of individual participant data. BMJ.

[9] Jat KR (2017). Vitamin D deficiency and lower respiratory tract infections in children: a systematic review and meta-analysis of observational studies. Trop Doct.

[10] Bergman P, Lindh AU, Bjorkhem-Bergman L, Lindh JD (2013). Vitamin D and respiratory tract infections: a systematic review and Meta-analysis of Randomized controlled trials. PLoS ONE.

[11] Mao S, Huang S (2013). Vitamin D supplementation and risk of respiratory tract infections: a meta-analysis of randomized controlled trials. Scand J Infect Dis.

[12] Charan J, Goyal JP, Saxena D, Yadav P (2012). Vitamin D for prevention of respiratory tract infections: a systematic review and meta-analysis. J Pharmacol Pharmacother.

[13] Salud. IdETe. Manual metodológico para la elaboración de evaluaciones de efectividad, seguridad y validez diagnóstica de tecnologías en salud. 2014.

[14] Salud INd. Informe de evento. Infeccion Respiratoria Aguda Bogota, Colombia: INS. ; 2017 [Available from: https://www.ins.gov.co/buscador-eventos/Informesdeevento/Informe%20IRA%20Final%202017.pdf.

[15] (DANE) DNdE. Archivo nacional de datos 2019 [Available from: https://sitios.dane.gov.co/anda-index/.

[16] (IHME) IfHMaE. Colombia Seattle. WA: University of Washington; 2021. [Available from: www.healthdata.org.

[17] Carroll AE, Downs SM (2009). Improving decision analyses: parent preferences (utility values) for pediatric health outcomes. J Pediatr.

[18] Husereau D, Drummond M, Petrou S, Carswell C, Moher D, Greenberg D (2013). Consolidated Health Economic evaluation reporting Standards (CHEERS) statement. Value Health.

[19] Moyano Ariza L. Estimación de costo-enfermedad por neumonía y bronquiolitis en niños menores de 5 años en Colombia. 2019.

[20] Ministerio de salud C. Sistema de Informacion de Precios de Medicamentos (SISMED) 2021 [03/07/2121]. Available from: www.sispro.gov.co/central-prestadores-de-servicios/Pages/SISMED-Sistema-de-Informacion-de-Precios-de-Medicamentos.aspx.

[21] Sakai M, Shimbo T, Omata K, Takahashi Y, Satomura K, Kitamura T (2008). Cost-effectiveness of gargling for the prevention of upper respiratory tract infections. BMC Health Serv Res.

[22] Berber A, Del-Rio-Navarro BE (2019). Cost-effectiveness analysis of OM-85 vs placebo in the prevention of acute respiratory tract infections (ARTIs) in children that attend day-care centers. Health Econ Rev.

[23] Zagar S, Lofler-Badzek D (1988). Broncho-Vaxom in children with rhinosinusitis: a double-blind clinical trial. ORL J Otorhinolaryngol Relat Spec.

[24] Pessey JJ, Megas F, Arnould B, Baron-Papillon F (2003). Prevention of recurrent rhinopharyngitis in at-risk children in France: a cost-effectiveness model for a nonspecific immunostimulating bacterial extract (OM-85 BV). PharmacoEconomics.

[25] Ravasio R (2015). Economic analysis of the immunostimulant OM-85 for the Prevention of Paediatric Recurrent Upper Respiratory Tract Infections. Global & Regional Health Technology Assessment.

[26] Hao Q, Dong BR, Wu T. Probiotics for preventing acute upper respiratory tract infections. Cochrane Database Syst Rev. 2015(2):CD006895. 10.1002/14651858.CD006895.pub3 25927096

[27] Chiappini E, Santamaria F, Marseglia GL, Marchisio P, Galli L, Cutrera R (2021). Prevention of recurrent respiratory infections: inter-society Consensus. Ital J Pediatr.

[28] Yin J, Xu B, Zeng X, Shen K (2018). Broncho-Vaxom in pediatric recurrent respiratory tract infections: a systematic review and meta-analysis. Int Immunopharmacol.

[29] Lassi ZS, Moin A, Bhutta ZA (2016). Zinc supplementation for the prevention of pneumonia in children aged 2 months to 59 months. Cochrane Database Syst Rev.

[30] Esposito S, Jones MH, Feleszko W, Martell JAO, Falup-Pecurariu O, Geppe N et al. Prevention of New Respiratory Episodes in Children with Recurrent Respiratory Infections: An Expert Consensus Statement. Microorganisms. 2020;8(11). 10.3390/microorganisms8111810 PMC7698530 33213053

[31] Cho HE, Myung SK, Cho H. Efficacy of Vitamin D Supplements in Prevention of Acute Respiratory Infection: A Meta-Analysis for Randomized Controlled Trials. Nutrients. 2022;14(4). 10.3390/nu14040818 PMC8879485 35215468

